# Content validity of the National Comprehensive Cancer Network – Functional Assessment of Cancer Therapy – Breast Cancer Symptom Index (NFBSI-16) and Patient-Reported Outcomes Measurement Information System (PROMIS) Physical Function Short Form with advanced breast cancer patients

**DOI:** 10.1186/s12955-019-1162-5

**Published:** 2019-05-29

**Authors:** Meaghan Krohe, Derek H. Tang, Brittany Klooster, Dennis Revicki, Nina Galipeau, David Cella

**Affiliations:** 1 Adelphi Values, 290 Congress St, 6th Floor, Boston, MA 02210 USA; 2 Novartis Oncology, One Health Plaza, 345/5130E, East Hanover, NJ 07936 USA; 3 Evidera, 7101 Wisconsin Avenue Suite 1400, Bethesda, MD 20814 USA; 4 Northwestern University, 633 N. St Clair, 19th Floor, Chicago, IL 60611 USA

**Keywords:** Oncology, Advanced breast cancer, Patient-reported outcomes, Health-related quality of life, Content validity, Qualitative research

## Abstract

**Background:**

The purpose of this study is to evaluate the content validity of the National Comprehensive Cancer Network – Functional Assessment of Cancer Therapy – Breast Cancer Symptom Index (NFBSI-16) and the Patient-Reported Outcomes Measurement Information System (PROMIS) Physical Function Short Form 10b among patients with hormone receptor positive (HR+)/human epidermal growth factor receptor 2 negative (HER2-) advanced breast cancer.

**Methods:**

Cognitive debriefing interviews sought to evaluate patients’ ability to read, understand, and meaningfully respond to the questionnaires, as well as to evaluate the questionnaires’ relevance in the target patient population. Interviews were conducted by telephone and lasted approximately 90 min. Audio recordings were transcribed, anonymized, and analyzed using qualitative data analysis software.

**Results:**

Fifteen cognitive debriefing interviews were conducted with women (mean age 66.0 years [standard deviation = 12.4]). Patients reported metastases in the bone (86.7%), liver (20.0%), lung (13.3%), skin (6.7%), and lymph node (6.7%) (not mutually exclusive). All patients for whom data were available demonstrated understanding of the instructions and the recall period of the NFBSI-16 (*n* = 14/14, 100.0%) and the PROMIS (*n* = 14/14, 100.0%). Greater than 90% of patients demonstrated understanding of each of the items in the NFBSI-16 and the PROMIS. Greater than 70% of patients demonstrated understanding of the response options of the NFBSI-16, > 90% understood response options of PROMIS Items 1–6, and ≥ 50% understood response options of PROMIS Items 7–10. Conceptual relevance was supported for most items in both questionnaires based on patients’ reports of experiencing the concepts as part of their breast cancer experience.

**Conclusions:**

The results of the cognitive debriefing interviews provide evidence that the NFBSI-16 and PROMIS Physical Function Short Form 10b have content validity in the HR+/HER2- advanced breast cancer patient population. Patients may benefit from additional instructions at the point the response options reverse direction in the PROMIS.

## Background

For patients with advanced breast cancer, both the disease process and its treatment give rise to numerous signs and symptoms that have negative impacts on their day-to-day lives (e.g., pain, skin irritation, etc.) [1]. Endocrine therapies are most commonly used in the treatment of hormone receptive-positive (HR+) advanced breast cancers, although recent studies suggest that quality of life is preserved longer in patients receiving combination therapies [2–5]. Common disease- and treatment-related symptoms experienced by patients with advanced breast cancer include pain and fatigue [6–10], which may be associated with more distal impacts on physical function such as decreased mobility and ability to carry out daily activities [6, 9, 11]. Few patient-reported outcome (PRO) questionnaires have been used to measure patient experience in advanced breast cancer subtypes.

In order to evaluate treatment benefits in advanced breast cancer, it is necessary to first understand the disease and treatment experience from the perspective of the patient. PRO measures can provide valuable insight into the patient experience, and their use in evaluating treatments in oncology has increased rapidly over the last several years. In considering the selection of a suitable PRO instrument to measure study endpoints, attention should be given to whether the instrument is content valid in the target patient population. Content validity refers to evidence that the PRO instrument measures concepts that are relevant to a disease and important to patients with that disease, and that the items are constructed in such a way that respondents can easily read and comprehend and to which they can provide meaningful responses [12, 13]. As stated in the United States Food and Drug Administration (FDA) *Guidance for Industry – Patient-Reported Outcomes Measures: Use in Medical Product Development to Support Labeling Claims,* such evidence can be established by conducting concept elicitation interviews with the target patient population to identify and describe the relevant and important concepts of a disease, and by conducting cognitive debriefing interviews with the target patient population to evaluate the comprehensibility, readability, and relevance of a PRO instrument [14].

In the current study, systematic reviews of the literature in advanced breast cancer and of available PRO instruments were conducted to identify potentially suitable PRO measures for use in an HR+/human epidermal growth factor receptor 2 negative (HER2-) advanced breast cancer population. Characteristics of the instruments of interest (including an evaluation of development histories and psychometric properties) and concepts that are considered directly related to disease status were assessed during the review [14]. As a result of these research activities, the National Comprehensive Cancer Network – Functional Assessment of Cancer Therapy – Breast Cancer Symptom Index (NFBSI-16) and the Patient-Reported Outcomes Measurement Information System (PROMIS) Physical Function Short Form 10b were selected as being most suitable to measure the important and relevant concepts of interest related to disease symptoms, treatment side effects, and physical functioning impacts in this patient population. The content validity of both the NFBSI-16 and PROMIS Physical Function Short Form 10b has been evaluated previously in breast cancer and cancer populations more generally [15–19], but not in an HR+/HER2- advanced breast cancer population specifically. Due to differences in disease trajectory and treatments among HR and HER2 subgroups, it is important to examine content validity in this specific subtype.

The purpose of this article is to describe the content evaluation of the PRO questionnaires (NFBSI-16 and PROMIS Physical Function Short Form 10b) through cognitive debriefing interviews with patients with HR+/HER2- advanced breast cancer.

## Methods

### Development history of measures

Cognitive debriefing interviews sought to evaluate patients’ ability to read, understand, and meaningfully respond to the questionnaires, as well as to evaluate the questionnaires’ overall relevance and ease of completion in the HR+/HER2- advanced breast cancer patient population. Prior to describing the cognitive debriefing methods for this study, we describe the development history (including any prior evaluation of the content validity or psychometric properties) of each instrument.

#### The National Comprehensive Cancer Network – Functional Assessment of Cancer Therapy – Breast Cancer Symptom Index (NFBSI-16)

The NFBSI-16 is a 16-item assessment of disease-related symptoms, treatment side effects, and general function and well-being. The instrument has three subscales: Disease-Related Symptom (DRS) – nine items; Treatment Side-Effect (TSE) – four items; and General Function and Well-Being (F/WB) – three items. All items have a seven-day recall period and a five-point verbal descriptive response scale [15].

The NFBSI-16 was developed as part of a larger project to create patient-reported symptom indexes for 11 different cancer types and builds upon the original Functional Assessment of Cancer Therapy (FACT) Breast Cancer Symptom Index (FBSI), and other components of the Functional Assessment of Chronic Illness Therapy (FACIT) measurement system [16]. Open-ended concept elicitation interviews were conducted with patients diagnosed with stage III or stage IV breast cancer (*N* = 52) to identify symptoms or concerns most important and relevant to them in their assessment of treatment value for their breast cancer [16]. Specifically, patients were first asked to generate a list of up to 10 symptoms and rank the importance of each symptom from 0 (“not important”) to 10 (“extremely important”) [16]. Additional concept elicitation interviews were conducted with patients diagnosed with stage III or stage IV breast cancer who had experience with chemotherapy (*N* = 52) to identify important and relevant concepts of their advanced breast cancer experience [16]. Patients also completed the FACT-Breast questionnaire [16]. Final item selection for the NFBSI-16 was driven by quantitative (i.e., frequency counts and measure of chance endorsement) and qualitative (i.e., review of the open-ended patient interviews) evaluation of the data [15]. Psychometric evaluations of the NFBSI-16 in advanced breast cancer populations, including convergent validity analyses and known-groups methods analyses, demonstrated acceptable measurement properties [15].

#### The Patient-Reported Outcomes Measurement Information System (PROMIS) Physical Function Short Form 10b

The PROMIS Physical Function Short Form 10b is a 10-item measure developed from the larger PROMIS physical function item bank of 124 items [20]. PROMIS defines the physical function latent trait as the ability to perform activities of daily living (ADLs; both general and instrumental) [21]. Items align with four subcategories: the functioning of one’s upper extremities (dexterity), lower extremities (walking or mobility), and central regions (neck, back), as well as instrumental ADLs (such as running errands) [22]. Items can assess more than one of these subcategories, but generally can be assigned to one predominant category [23]. Items are meant to be answered using the present tense and have a five-point verbal rating scale.

The PROMIS Physical Function Short Form 10b was developed consistent with all PROMIS item banks and for use across disease areas [24, 25]. Further research activities were conducted to examine applicability to cancer populations. Content analysis of data from a diverse sample of patients with cancer in focus groups (*N* = 21) and cognitive interviews (*N* = 40) was used to inform domain experts’ qualitative item review. Item modifications were made to reflect cancer-specific concerns (for example, adding items regarding neuropathic pain, which was supported by data analysis and expert consensus) [17]. Similar versions of the PROMIS Physical Functioning Short Form were shown to have acceptable measurement properties (including internal consistency reliability, test-retest reliability, and construct-related validity) among advanced solid tumor populations (including breast cancer) [18, 19].

### Patient recruitment

The patients who were recruited for this study, following independent review board approval, were identified at four clinical sites located in New Orleans, Louisiana (*n* = 10); St. Louis, Missouri (*n* = 3); Chicago, Illinois (*n* = 1); and Detroit, Michigan (*n* = 1). Clinicians confirmed patients’ diagnosis of HR+/HER2- advanced breast cancer, among other inclusion and exclusion criteria (Table 1), prior to patients being included in the study.

**Table 1:** Study inclusion/exclusion criteria

Inclusion criteria: • Subject had to sign and date a written Informed Consent Form (ICF); • Subject was an adult man or woman (≥18 years of age) at the time of screening; • Subject had clinician confirmation of metastatic or locally advanced breast cancer not amenable to curative treatment by surgery or radiotherapy; • Subject had clinician confirmation of hormone-receptor positive (HR+; estrogen receptor positive and/or progesterone receptor positive) and human epidermal growth factor receptor-2 negative (HER2-) breast cancer; • Subject was able to speak, read, write, and comprehend US English fluently, as determined by clinician; and • Subject was willing and able to participate in a 90-min, face-to-face or telephone interview. Exclusion criteria: • Subject had received more than three lines of therapy for advanced breast cancer; • Subject had central nervous system involvement such as brain metastases or spinal cord compression; • Subject had participated in an interventional clinical trial in the past 30 days; or • Subject had any other concurrent severe and/or uncontrolled medical condition including a cognitive impairment or disorder that might confound study results and/or contraindicate subject’s participation in the study, in the opinion of the clinician.	

### Conduct of interviews

The cognitive debriefing interviews were conducted with patients one-on-one, over the telephone, by trained researchers. Each interview lasted approximately 90 min and was audio recorded with the subject’s prior written and verbal consent. The questionnaires were cognitively debriefed with patients following a concept elicitation exercise; the concept elicitation portion of the interviews has been reported elsewhere. The cognitive debriefing portion of the interview lasted approximately one hour.

Patients were provided electronic copies of both the NFBSI-16 and PROMIS just prior to the interview. While patients were cognitively debriefed on both questionnaires during the same interview, the order in which they were debriefed was rotated so that the same questionnaire was not debriefed first in all interviews. During the interview, patients were first asked to complete the questionnaires using a “think aloud” method. This method allowed patients the opportunity to complete each questionnaire without any interruption from the interviewer, and to describe aloud how they arrived at each answer, which helps to identify words, terms, or concepts that they may not understand or might interpret differently than intended [26]. After the patient completed the first questionnaire using the “think aloud” method, the interviewer then followed a semi-structured cognitive debriefing interview guide to elicit specific feedback on that questionnaire that might not have been covered during the think-aloud process. For example, the following types of questions were asked by the interviewer to assess patient understanding after the think-aloud exercise:

- What does this [instruction/question/response option] mean to you?
- Can you put this [instruction/question/response option] into your own words?
- What made you choose [response selected by subject]?

In addition, the interviewer asked about the questionnaires’ relevance and overall ease or difficulty of completion. After cognitive debriefing was completed on the first questionnaire, the same process was followed for the second questionnaire.

### Coding and analysis

Interviews were audio-recorded, transcribed, and anonymized (i.e., identifying information was removed). Transcripts were imported into a computerized qualitative data analysis package to facilitate the storing, coding and retrieval of qualitative data using Boolean operators [27]. A codebook was developed based on the components of the questionnaires and interview questions. For example, codes were created to tag patient quotes representing their interpretation of Item 1 of the NFBSI-16 and to distinguish those patients who interpreted the item as intended from those who did not: “NFBSI::Item 1::Interpreted as intended::Yes” or “NFBSI::Item 1:: Interpreted as intended::No.” A similar coding structure was followed for each component of the questionnaires and for each type of interview question.

The research team reviewed patient quotes as they related to the study objectives. Patients’ interpretations of each instruction, item, and response option in the questionnaires were evaluated, and a determination was made by the research team as to whether each patient demonstrated understanding of each component of the questionnaire per the criteria described in the study protocol and consistent with recommended practices [13].

Results were tabulated, summarized, and presented to support the content validity of the questionnaires and to identify potential areas for improvement in measurement consistent with recommended practices [13].

All frequencies and percentages reported were based on the number of patients who provided sufficient data that could be used in analysis; some data were not collected from every patient, or responses may have been insufficient or uninterpretable as determined by the research team. Therefore, total frequency and percent calculations are reported based on the number of patients for whom data were available, and not necessarily the total sample of 15.

## Results

### Study sample

All 15 patients were women, with an age range from 45.3 to 87.6 years (mean = 66.0, standard deviation = 12.4). The majority were white (*n* = 8/15, 53.3%), and the greatest number had attended some college or certificate program (*n* = 7/15, 46.7%) and were retired (*n* = 7/15, 46.7%) at the time of the interview. Clinically, the majority of patients were post-menopausal (*n* = 12/15, 80%), had breast cancer metastasized to the bone (*n* = 13, 80.0%), and had an Eastern Cooperative Oncology Group (ECOG) score of 1 (*n* = 11, 73.3%). Full patient demographic and health information can be found in Tables 2 and 3.

**Table 2:** Patient-reported demographic and health information

	Total (*N* = 15) *n* (%)^a^
Age (years)	
Range	45.3–87.6
Mean (standard deviation)	66.0 (12.4)
Gender	
Female^b^	15 (100.0%)
Spanish/Hispanic/Latino ethnicity	
Not Spanish/Hispanic/Latino	14 (93.3%)
Mexican/Mexican American, Chicano	1 (6.7%)
Race	
Black or African American	6 (40.0%)
White/Caucasian	8 (53.3%)
Other	1 (6.7%)
Education	
High school diploma (or GED) or less	0 (0.0%)
Some college or certificate program	7 (46.7%)
College or university degree (two- or four-year)	5 (33.3%)
Graduate degree	1 (6.7%)
Other	2 (13.3%)
Living status	
Living with family or friends	13 (86.7%)
Living alone	2 (13.3%)
Annual household income	
Under $25,000	1 (6.7%)
$25,000 to $49,999	6 (40.0%)
$50,000 to $74,999	3 (20.0%)
$75,000 to $99,999	1 (6.7%)
$100,000 and over	1 (6.7%)
Prefer not to answer	3 (20.0%)
Work status^c^	
Working full-time	4 (26.7%)
Working part-time	2 (13.3%)
Homemaker	1 (6.7%)
Retired	7 (46.7%)
Unemployed	1 (6.7%)
Receiving disability benefits	1 (6.7%)
Health in general	
Excellent	0 (0.0%)
Very good	0 (0.0%)
Good	8 (53.3%)
Fair	5 (33.3%)
Poor	2 (13.3%)
Other health conditions^c^	
Heart disease	1 (6.7%)
High blood pressure	8 (53.3%)
High cholesterol	2 (13.3%)
Pain	6 (40.0%)
*Muscle pain*	4 (26.7%)
*Neuropathic pain*	2 (13.3%)
*Other pain*	1 (6.7%)
Diabetes	2 (13.3%)
*Type 2*	*2 (13.3%)*
Thyroid disease	1 (6.7%)
Depression/anxiety	6 (40.0%)
None	2 (13.3%)
Other	4 (26.7%)

^a^Unless other statistic indicated

^b^Although male patients were eligible, none were identified at any recruitment site

^c^Not mutually exclusive

**Table 3:** Clinician-reported health information

	Total (*N* = 15) *n* (%)
Menopausal status	
Pre-menopausal	3 (20.0%)
*Not on gonadotropin-releasing hormone (GnRH) agonist treatment*	*3 (100.0%)*
Post-menopausal	12 (80.0%)
Mammalian target of rapamycin (mTOR) inhibitor treatment	
Yes	3 (20.0%)
No	12 (80.0%)
Recurrent or progressive disease refractory to non-steroidal aromatase inhibitor (NSAI), tamoxifen, or fulvestrant	
Yes	6 (40.0%)
No	9 (60.0%)
Eastern Cooperative Oncology Group (ECOG) score	
0	1 (6.7%)
1	11 (73.3%)
2	3 (20.0%)
Cyclin-dependent kinase (CDK4/6) inhibitor treatment	
Yes	5 (33.3%)
No	10 (66.7%)
Metastatic site^a^	
Bone	13 (86.7%)
Lung	2 (13.3%)
Liver	3 (20.0%)
Lymph node	1 (6.7%)
Skin	1 (6.7%)

^a^Not mutually exclusive

### Cognitive debriefing interview results

#### National Comprehensive Cancer Network – Functional Assessment of Cancer Therapy – Breast Cancer Symptom Index (NFBSI-16)

All patients for whom data were available (*n* = 14/14, 100.0%) demonstrated understanding of the instructions and the recall period of the NFBSI-16. Overall, greater than 90% of patients demonstrated understanding of each of the item questions. Specifically, all patients who provided sufficient data (*n* ≥ 12) demonstrated complete (100%) understanding of 14 of the 16 items of the NFBSI-16. For the remaining two items, 14 out of 15 patients (93.3%) demonstrated understanding of Item 3 (feeling ill), and 11 out of 12 patients (91.7%) demonstrated understanding of Item 10 (nausea). Additionally, greater than 70% of patients for whom data were available demonstrated understanding of all of the response options: “Not at all” (*n* = 12/13, 92.3%), “A little bit” (*n* = 10/14, 71.4%), “Somewhat” (*n* = 11/11, 100.0%), “Quite a bit” (n = 11/13, 84.6%), and “Very much” (*n* = 9/11, 81.8%). In terms of item relevance and comprehensiveness, ≥80% of patients reported experiencing at least 11 out of the 16 concepts as a part of the HR+/HER2- advanced breast cancer experience (lack of energy, pain, feeling ill, shortness of breath, family role, fatigue, pain, worry, ability to work, ability to enjoy life, and quality of life), while between 42 and 67% of patients endorsed the remaining five concepts (bone pain, nausea, side effects, hair loss, mouth sores). Overall, the majority of patients reported that they did not believe additional concepts should be added to the questionnaire and that the questionnaire was easy to complete (*n* = 11/14, 78.5% and *n* = 12/13, 92.3%, respectively) (Table 4).

**Table 4:** NFBSI-16 cognitive debriefing summary Table (*N* = 15)

Item, instruction, or response option	Number of patients (*n*,%)^a^	Exemplary quotes demonstrating patient understanding of instruction or item concept
Instructions: Below is a list of statements that other people with your illness have said are important. Please circle or mark one number per line to indicate your response as it applies to the past 7 days.		
Item was understood by patient	14/14 (100%)	*“To read the question and circle number zero through four depending on which pertains to the activity you’ve had in the last week.”*
Concept was experienced by patient	–	
Item 1: I have a lack of energy		
Item was understood by patient	13/13 (100.0%)	*“… I am very active, but my energy level is not where it was before I started going through this whole process dealing with, um, cancer. So that’s why I pick a little bit because even during the regular day I don’t feel that I’m at 100%. I’m feeling a little low on the energy.”*
Concept was experienced by patient	15/15 (100.0%)	
Item 2: I have pain		
Item was understood by patient	14/14 (100.0%)	*“And that was because I was telling you, um, under my left arm from the lymph nodes I always have pain there. It’s not really bad. It’s probably like a three. And I have pain in my injection spots. And that lasts throughout the whole month almost. So I still have pain or soreness in those areas. So that’s why I picked a little bit.”*
Concept was experienced by patient	12/15 (80.0%)	
Item 3: I feel ill		
Item was understood by patient	14/15 (93.3%)	*“Just not feeling well. You know, um, just feeling sick.”*
Concept was experienced by patient	12/14 (85.7%)	
Item 4: I have been short of breath		
Item was understood by patient	15/15 (100.0%)	*“I lose my breath quite a bit when I go up and down the stairs.… Or I walk outside. … Phew, it takes your breath away.”*
Concept was experienced by patient	12/14 (85.7%)	
Item 5: Because of my physical condition, I have trouble meeting the needs of my family		
Item was understood by patient	15/15 (100.0%)	*“Uh, it’s just my husband and I so, um, uh, it’s difficult because I can’t do what I used to do. You know, you have to depend on people. Um, but I try to do what I can.… Yeah. ‘Cause I push myself.… So I’m going to say somewhat.… getting stuff ready. Even though he cooks, you know, I will try to fix the salad or get things ready. Get our plates ready and sometimes that’s difficult.”*
Concept was experienced by patient	13/14 (92.9%)	
Item 6: I feel fatigued		
Item was understood by patient	14/14 (100.0%)	*“Q: And what does fatigue mean to you? A: Drained, tired, achy, lethargic.”*
Concept was experienced by patient	14/14 (100.0%)	
Item 7: I have bone pain		
Item was understood by patient	12/12 (100.0%)	*“I would say if you had bone pain, that means the cancer has gotten into your bones and your bones are actually aching and cancer hasn’t gotten into my bones …”*
Concept was experienced by patient	8/14 (57.1%)	
Item 8: I am sleeping well		
Item was understood by patient	15/15 (100.0%)	*“And I answered that like that because, um, sometimes when I go to sleep I wake up in the middle of the night and I can’t go back to sleep. And then sometimes, um, even when I’m feeling tired like I’m sleepy, sometimes I can’t even go to sleep. So I might just stay up and try to watch TV until my body just decides to doze off. So that’s what I mean, but I, I’m really not sleeping well. Even when I’m – when I am asleep it’s like I can literally feel myself tossing and turning. It’s not like a just relaxed sleep.”*
Concept was experienced by patient	12/14 (85.7%)	
Item 9: I worry that my condition will get worse		
Item was understood by patient	14/14 (100.0%)	*“Because I’ve had it and it hasn’t gotten any worse. So it could get worse, but it’s not something to worry about right now. Q: Okay. Um, and when – let’s see, when it says worry my condition will get worse, what does that mean to you? A: That I constantly think about it, which I don’t.”*
Concept was experienced by patient	12/14 (85.7%)	
Item 10: I have nausea		
Item was understood by patient	11/12 (91.7%)	*“That I want to throw up. That you feel like throwing up…”*
Concept was experienced by patient	9/14 (64.3%)	
Item 11: I have mouth sores		
Item was understood by patient	14/14 (100.0%)	*“Um, like canker sores or something that maybe the treatment has caused a, a soreness or an open wound in your mouth.”*
Concept was experienced by patient	6/14 (42.9%)	
Item 12: I am bothered by side effects		
Item was understood by patient	14/14 (100.0%)	*“I am bothered, uh, somewhat, I wish they would go away.… Side effects, um, side effects of the treatments. Examples, uh, stiffness, vomiting, uh, hair loss, those are side effects of the treatment that I have taken.”*
Concept was experienced by patient	10/15 (66.7%)	
Item 13: I am bothered by hair loss		
Item was understood by patient	14/14 (100.0%)	*“I am bothered by hair loss. Not really. Not at all because I was told that it would happen. Q: Okay. Um, and what does, what does hair loss mean to you? A: That my hair would fall out.”*
Concept was experienced by patient	9/14 (64.3%)	
Item 14: I am able to work (include work at home)		
Item was understood by patient	15/15 (100.0%)	*“Um, going to your job that – your employment, um, or working at home would be just general chores, um, housework, cooking, things of that nature.”*
Concept was experienced by patient	15/15 (100.0%)	
Item 15: I am able to enjoy life		
Item was understood by patient	15/15 (100.0%)	*“I’m beyond everything right now as far as I don’t let the aches and pains bother me. I mean I’ll grumble about them, but I’ll get past them. So for me that’s a four. Q: And what does enjoy life mean to you? A: Getting out and doing what you want to do or not letting someone tell you, you can’t do it because you had cancer or trying to – in their eyes being helpful, but in your eyes kind of not allowing you to do something just let you try it.”*
Concept was experienced by patient	11/12 (91.7%)	
Item 16: I am content with the quality of my life right now		
Item was understood by patient	15/15 (100.0%)	*“Quality of life, being able to get around without anybody, with aid, that’s a quality of life, to live the life you lived before chemo. … I’m content with and always say it is more important to have a quality of life instead of a quantity of it. So if, ah, all went bad and I, and cancer would take over, ah, I would have that to, ah, evaluate it and say what about quantity, living and not being able to do, have, to do the things I want to do.”*
Concept was experienced by patient	11/12 (91.7%)	
Response options		
Response option “Not at all” understood by patient	12/13 (92.3%)	–
Response option “A little bit” understood by patient	10/14 (71.4%)	–
Response option “Somewhat” understood by patient	11/11 (100.0%)	–
Response option “A lot” understood by patient	11/13 (84.6%)	–
Response option “Very much” understood by patient	9/11 (81.8%)	–
Overall		
Patient reported NFBSI-16 is easy to complete	12/13 (92.3%)	–
Patient reported that there are no missing concepts	11/14 (78.6%)	

^a^Note: The total counts vary based on the number of patients who provided sufficient data that could be used in analysis

#### Patient-Reported Outcomes Measurement Information System (PROMIS) Physical Function Short Form 10b

All patients for whom data were available (*n* = 14/14, 100.0%) interpreted the instructions of the PROMIS as intended. Overall, greater than 70.0% of patients demonstrated understanding of each item as intended. Specifically, all patients who provided sufficient data (*n* ≥ 11) demonstrated complete understanding (100%) of eight of the 10 PROMIS items. For the remaining two items, 13 out of 14 patients (92.9%) demonstrated understanding of Item 7 (vigorous physical activities), and 11 out of 12 patients (91.7%) demonstrated understanding of Item 9 (putting trash outside).

Additionally, two sets of response options were debriefed. The first set of response options, “Without any difficulty, With a little difficulty, With some difficulty, With much difficulty, Unable to do,” used for Items 1–6, were understood by > 90% of patients for whom data were available (*n* ≥13). The second set of response options was less well understood by patients; four of the five response options were interpreted as intended by ≥50% of patients for whom data were available (*n* ≥ 10) (“Not at all, Somewhat, Quite a lot, and Cannot do”), and only 25.0% for whom data were available (*n* = 12) demonstrated understanding of the response option “Very little.” In terms of item relevance, ≥75.0% of patients reported at least nine out of the 10 concepts as part of their HR+/HER2- advanced breast cancer experience (ability to do chores, ability to go up and down stairs, ability to run errands or shop, ability to bend down, ability to lift weight above shoulders, vigorous physical activities, bathing or dressing, moderate physical activities, and ability to get in and out of car), while 57.1% of patients endorsed the remaining concept (putting trash outside). Additionally, while the PROMIS has no stated recall period, almost all patients (*n* = 13, 86.7%) answered the items based on their current status. Overall, the majority of patients (*n* = 11/13, 84.6%) did not believe additional concepts should be added to the questionnaire. All of the patients (*n* = 15/15, 100.0%) indicated that the questionnaire was easy to complete (Table 5).

**Table 5:** PROMIS Physical Function Short Form 10b cognitive debriefing summary Table (*N* = 15)

Item, instruction, or response option	Number of patients (*n*,%)^a^	Exemplary quotes demonstrating patient understanding of instruction or item concept
Instructions: Please respond to each question or statement by marking one box per row.		
Item was understood by patient	14/14 (100%)	*“It says read each question or the statement and mark, and marking one box per row. It means it’s asking me to read the question and mark one box on my answers.”*
Concept was experienced by patient	–	
Item 1: Are you able to do chores such as vacuuming or yard work?		
Item was understood by patient	15/15 (100.0%)	*“Unable to do, number 1. Q: Okay, and why do you choose unable to do? A: Because vacuuming, I can vacuum only if I’m in my wheelchair or walker, and then yard work, it is too hot to do yard work.… Chores, ah, washing dishes, cooking, vacuuming, mopping, dish, ah, laundry.”*
Concept was experienced by patient	14/15 (93.3%)	
Item 2: Are you able to get in and out of a car?		
Item was understood by patient	14/14 (100.0%)	*“Okay, like if you go to the grocery store, can you get in that car? Can you put all your groceries in your car, can you get in without need assistance? I don’t need any assistance to get in and I don’t need any assistance to get out.”*
Concept was experienced by patient	11/14 (78.6%)	
Item 3: Are you able to go up and down stairs at a normal pace?		
Item was understood by patient	15/15 (100.0%)	*“Are you able to go up and down stairs at a normal pace? Um, going down is okay. Going up, it’s very difficult. Q: What is it about going up that is difficult? A: I can’t breathe. Q: … in this question what does a normal pace mean to you? A: Just walking at a, um, normal speed.”*
Concept was experienced by patient	13/15 (86.7%)	
Item 4: Are you able to run errands and shop?		
Item was understood by patient	13/13 (100.0%)	*“Run errands and shop, uh, go to the cleaners, put gas in their car, go do this, go do that, and do the groceries. You just don’t have it in you.”*
Concept was experienced by patient	12/15 (80.0%)	
Item 5: Are you able to bend down and pick up clothing from the floor?		
Item was understood by patient	15/15 (100.0%)	*“We’re going to go with some difficulty on that depending on how far away the floor is. I actually have little grabbers that I keep in different rooms of the house so that I can reach down and grab things with.… if I have to physically bend down and pick it up it’s more of a challenge for me to grab it and get up. Um, if it’s rounded up and if I can just reach down a little easier I can grab it that way. But if it’s something flat on the floor it’s just a, a nuisance for me. It’s, it’s kind of midrange. I can do it, but do I want to do it? No.”*
Concept was experienced by patient	12/14 (85.7%)	
Item 6: Are you able to lift 10 pounds (5 kg) above your shoulder?		
Item was understood by patient	11/11 (100.0%)	*“Either pick up from a table or something that’s handed to me and then raise it up maybe to a shelf or, or move it from one position to another that really wouldn’t be above my shoulders but, um, just moving that object from where it was to where it’s going to. Not necessarily like a bench press or something like that like raising weights.”*
Concept was experienced by patient	11/12 (91.7%)	
Item 7: Does your health now limit you in doing vigorous activities, such as running, lifting heavy objects, participating in strenuous sports?		
Item was understood by patient	13/14 (92.9%)	*“I cannot run, I cannot lift anything, and I cannot participate in sports, so I cannot do any of that.”*
Concept was experienced by patient	13/15 (86.7%)	
Item 8: Does your health now limit you in bathing or dressing yourself?		
Item was understood by patient	13/13 (100.0%)	*“Because you need help with helping wash your back and sometimes pulling up pants or zipping objects.”*
Concept was experienced by patient	13/14 (92.9%)	
Item 9: Does your health now limit you in putting a trash bag outside?		
Item was understood by patient	11/12 (91.7%)	*“Can you lift that trash bag out of that trash can and take it to the dumpster or take it to the big trashcan outside, the garbage can.”*
Concept was experienced by patient	8/14 (57.1%)	
Item 10: Does your health now limit you in doing moderate activities, such as moving a table, pushing a vacuum cleaner, bowling, or playing golf?		
Item was understood by patient	15/15 (100.0%)	*“I don’t bowl or play golf. I couldn’t get in the heat or that kind of weather or anything. I don’t do any moderate activities. Moving a table, no, I would never attempt that.… Um, so there’s only one in there I would do.… With some difficulty, the vacuum.… Yeah. I couldn’t pick up a bowling ball and golf I wouldn’t get out in the heat or the sun.… And, um, picking up a table, I’m not going to pick up a table either. I don’t have the strength for that.”*
Concept was experienced by patient	10/12 (83.3%)	
Response options		
Response option “Without any difficulty” understood by patient	15/15 (100.0%)	–
Response option “With some difficulty” understood by patient	13/13 (100.0%)	–
Response option “With much difficulty” understood by patient	15/15 (100.0%)	–
Response option “Unable to do” understood by patient	15/15 (100.0%)	–
Response option “Not at all” understood by patient	7/14 (50.0%)	–
Response option “Very little” understood by patient	3/12 (25.0%)	–
Response option “Somewhat” understood by patient	8/10 (80.0%)	–
Response option “Quite a lot” understood by patient	6/10 (60.0%)	–
Response option “Cannot do” understood by patient	13/13 (100.0%)	–
Overall		
Patient reported PROMIS is easy to complete	15/15 (100.0%)	–
Patient reported that there are no missing concepts	11/13 (84.6%)	

^a^Note: The total counts vary based on the number of patients who provided sufficient data that could be used in analysis

## Discussion

For the NFBSI-16, cognitive debriefing results showed strong support for the content validity of the measure in a HR+/HER2- advanced breast cancer population; all of the patients in the sample for whom data were available understood the instructions and the majority of the items (14 out of 16), while the remaining two items were understood by at least 90% of the patient sample. Further, the majority of the concepts measured by the NFBSI-16 were relevant to at least 80% of the sample, and all of the concepts were relevant to at least 40%. Similarly, at least 90% of the patient sample demonstrated understanding of the instructions and items of the PROMIS Physical Function Short Form 10b. Almost all of the concepts were relevant to at least 75% of the sample. The cognitive debriefing results for the NFBSI-16 and PROMIS Physical Function Short Form 10b demonstrated that HR+/HER2- advanced breast cancer patients were able to complete and comprehend the questionnaires in ways consistent with developer and researcher expectations, and that overall, the concepts covered by the questionnaires are relevant to the patient experience of HR+/ HER2- advanced breast cancer.

While some patients did not utilize the response options as expected for some items in the PROMIS, they demonstrated understanding of the item-concepts and the response scale overall. For example, Item 7 asked “Does your health now limit you in doing vigorous activities…?” and a subject selected “Not at all” with the rationale that she could not do vigorous activities at all, though the correct response option for her ability would have been “Cannot do.” The misunderstanding of the response scale may potentially have been a result of the negatively worded items, which switched direction from the previous items in the questionnaire and may be more prone to response error. This may also explain why the response options “Very little” and “Quite a lot” were less well understood by patients (*n* = 3/12, 25.0% and *n* = 6/10, 60.0%, respectively); however, the response option “Somewhat” was well understood (*n* = 8/10, 80.0%), as middle response options may be understood regardless of response scale direction. Another possible explanation could be that both ends of the response scale have negatively worded anchors (i.e., “Not at all” and “Cannot do”), which may have contributed to some of the misinterpretation.

In general, if a majority of patients understand the content as it is intended, there is justification to leave the questionnaire as is. Despite cognitive interviews being a widely used method, there are various approaches to interpreting data. For instance, there are no established and agreed upon thresholds for determining whether modifications are needed to a PRO instrument based on cognitive interview results. Some studies, such as the current one, do not employ any a priori threshold, and modifications might be deemed beneficial and necessary even though the majority demonstrated understanding. In its paper on establishing content validity, the International Society For Pharmacoeconomics and Outcomes Research (ISPOR) Task Force acknowledges the lack of clarity around whether modifications to an instrument are warranted if only a minority of patients misinterpret the content [13]. Ultimately, the decision to make any modifications should be made in consideration of whether the proposed change would increase the overall content validity of the instrument. In the current study, additional instructions before the second set of response options in the PROMIS Physical Function Short Form 10b could be one suggestion to help orient the respondent to the change in the directional nature of the items and response scale, and potentially help alleviate response error.

The limitations of the study center on the clinical and demographic characteristics of the patient sample, which may impact generalizability of results. Findings on the content validity of the questionnaires may not be generalizable to other subtypes of breast cancer or be comprehensive across all treatment experiences or tumor types, alongside the fact that the more severely ill may be less likely to participate in the study. In addition, all patients in this sample had completed some college, and most were treated at a single center in Louisiana.

## Conclusions

The results of the cognitive debriefing interviews provide evidence that the NFBSI-16 and PROMIS Physical Function Short Form 10b assess the disease-related symptoms, treatment-related side effects, and physical functioning impact concepts that are important and relevant to HR+/HER2- advanced breast cancer patients, and do so in ways that patients can understand and to which they can meaningfully respond. Additional instructions on the PROMIS Physical Function Short Form 10b to orient the respondent to the response scale direction may increase respondent understanding. These findings add to the evidence of content validity of the NFBSI-16 and PROMIS Physical Function Short Form 10b in an advanced breast cancer patient population. Future research should further confirm the questionnaires as being “fit for purpose” in the target patient population via psychometric evaluation and score interpretation in regulated clinical trials.

## Data Availability

The datasets generated and/or analyzed during the current study are generally available from the corresponding author upon reasonable request. However, portions of the content may not be available due to confidentiality reasons.

## Abbreviations

ADLs: activities of daily living
DRS: Disease-Related Symptom
ECOG: Eastern Cooperative Oncology Group
F/WB: General Function and Well-Being
FACIT: Functional Assessment of Chronic Illness Therapy
FACT: Functional Assessment of Cancer Therapy
FBSI: Functional Assessment of Cancer Therapy Breast Cancer Symptom Index
HER2-: human epidermal growth factor receptor 2
HR +: hormone receptive-positive
ISPOR: International Society for Pharmacoeconomics and Outcomes Research
NFBSI-16: National Comprehensive Cancer Network – Functional Assessment of Cancer Therapy – Breast Cancer Symptom Index
PRO: patient-reported outcome
PROMIS: Patient-Reported Outcomes Measurement Information System
TSE: Treatment Side Effect
